# Incidence of catheter-related bloodstream infections following ultrasound-guided central venous catheterization: a systematic review and meta-analysis

**DOI:** 10.1186/s12879-022-07760-1

**Published:** 2022-10-04

**Authors:** Jun Takeshita, Kazuya Tachibana, Yasufumi Nakajima, Nobuaki Shime

**Affiliations:** 1 Department of Anesthesiology, Osaka Prefectural Hospital Organization, Osaka Women’s and Children’s Hospital, Osaka, Japan; 2 Department of Anesthesiology and Intensive Care, Kinki University Faculty of Medicine, Osaka, Japan; 3 Outcomes Research Consortium, Cleveland, OH USA; 4 Department of Emergency and Critical Care Medicine, Institute of Biomedical & Health Sciences, Hiroshima University, Hiroshima, Japan

**Keywords:** Central venous catheterization, Ultrasonography, Bloodstream infections, Incidence

## Abstract

**Background:**

Ultrasonographic guidance is widely used for central venous catheterization. Several studies have revealed that ultrasound-guided central venous catheterization increases the rate of success during the first attempt and reduces the procedural duration when compared to the anatomical landmark-guided insertion technique, which could result in protection from infectious complications. However, the effect of ultrasound-guided central venous catheterization on catheter-related bloodstream infections remains unclear. We aimed to conduct a systematic review and meta-analysis to evaluate the value of ultrasound guidance in preventing catheter-related bloodstream infections and catheter colonization associated with central venous catheterization.

**Methods:**

The Cochrane Central Register of Controlled Trials (CENTRAL) and MEDLINE (via PubMed) were searched up to May 9, 2022 for randomized controlled trials (RCTs) comparing ultrasound-guided and anatomical landmark-guided insertion techniques for central venous catheterization. Risk of bias was assessed using the Cochrane Risk of Bias 2 (RoB 2) tool for RCTs. A meta-analysis was performed for catheter-related bloodstream infections and catheter colonization, as primary and secondary outcomes, respectively.

**Results:**

Four RCTs involving 1268 patients met the inclusion criteria and were analyzed. Ultrasound-guided central venous catheterization was associated with a slightly lower incidence of catheter-related bloodstream infections (risk ratio, 0.46; 95% confidence interval [CI], 0.16–1.32) and was not associated with a lower incidence of catheter colonization (risk ratio, 1.36; 95% CI, 0.57–3.26).

**Conclusion:**

Ultrasound-guided central venous catheterization might reduce the incidence of catheter-related bloodstream infections. Additional RCTs are necessary to further evaluate the value of ultrasound guidance in preventing catheter-related bloodstream infections with central venous catheterization.

**Supplementary Information:**

The online version contains supplementary material available at 10.1186/s12879-022-07760-1.

## Background

The ultrasound-guided insertion technique for central venous catheterization is widely used, as it is reported to increase the success rate and decrease the rate of mechanical complications such as arterial mispuncture, pneumothrax, and hematoma when compared to the anatomical landmark-guided insertion technique [1–3].

Catheter-related bloodstream infections (CRBSIs) are serious complications associated with central venous catheterization. They can result in increased costs and risk of mortality [4–7]. The incidence of CRBSIs was reported to be 2.2 to 2.79 infections per 1000 catheter days [8, 9]. An increase in the success rate of the first attempt and shortening of the procedural duration by ultrasound guidance can result in protection from contamination of catheters as well as the insertion site during insertion, and ultrasound-guided central venous catheterization is recommended for preventing CRBSIs in pediatric intensive care units [10]. Furthermore, several guidelines also recommend the use of ultrasonography, which minimizes contamination by reducing the number of attempts and breakdown of the aseptic technique and decreases the rate of CRBSIs in adults and children [11–14]. However, the clinical evidence remains unclear.

A recent post hoc analysis of three randomized controlled trials demonstrated that the ultrasound-guided insertion technique was associated with an increased risk of CRBSIs [15]. However, the patients were not randomized according to the insertion technique. Therefore, the association between ultrasound-guided central venous catheterization and CRBSIs remains unclear. Ultrasound guidance may reduce the number of attempts but may increase contamination during the process of manipulation. Although ultrasound-guided central venous catheterization is mandatory today, we hypothesized that it would be worthwhile to evaluate the efficacy of ultrasound guidance on the incidence of CRBSIs. Hence, we conducted a systematic review and meta-analysis to determine the value of ultrasound guidance in preventing CRBSIs and catheter colonization associated with central venous catheterization.

## Methods

### Search strategy

This systematic review was reported according to the PRISMA guidelines [16] and was based on the methodology recommended by the Cochrane Handbook for Systematic Reviews of Interventions version 6.3 (updated February 2022). The protocol of the study was registered with PROSPERO (registration No. CRD 42,022,319,649).

We searched MEDLINE (via PubMed) and the Cochrane Central Register of Controlled Trials (CENTRAL) databases for relevant randomized controlled trials up to May 9, 2022. We used the following search terms, with the language restricted to English: (“ultrasound” or “ultrasonography” or “ultrasonographically”) and (“central venous catheter”) and (“randomized controlled trial” or “controlled clinical trial” or “randomized” or “placebo” or “randomly” or “trial” or “group”). The inclusion criteria were as follows: studies involving patients (P) who underwent central venous catheterization; studies in which the intervention (I) was ultrasound-guided central catheter insertion; studies in which the control (C) was the anatomical landmark-guided insertion technique; and studies with CRBSIs and catheter colonization as outcomes (O).

### Study selection

Following the removal of duplicates using the Rayyan QCRI software (https://rayyan.ai/), two authors reviewed the titles and abstracts independently and assessed the eligibility of various manuscripts. Then, full texts were assessed for eligibility. Any disagreement was resolved by discussion, and a consensus was reached.

### Data collection and quality assessment: risk of bias assessment and GRADE approach

The following data were extracted by two authors independently: First author, year of publication, country, age of patients, sample size, and outcome. Using the Cochrane Risk of Bias 2 (RoB 2) tool for RCTs [17], the risk of bias was assessed by two authors independently. The included trials were classified as having a low risk of bias, some concern, or a high risk of bias. The quality of evidence of each outcome was graded according to the criteria established by the GRADE working group [18]. Any disagreement was settled through discussion until a consensus was reached.

### Data analyses

The primary outcome of this systematic review was incidence of CRBSIs. The secondary outcome was catheter colonization. We defined CRBSIs and catheter colonization according to the Infectious Diseases Society of America [19]. All data analyses were executed using Review Manager version 5.4.1 (Cochrane Collaboration, Oxford, UK). The weighted treatment effect was calculated across trials. Results were expressed as risk ratios (RR) with 95% confidence intervals (CI) for dichotomous outcomes. All reported p-values were two-sided, and p-values < 0.05 were considered statistically significant. We evaluated heterogeneity using the I^2^ test, and significant heterogeneity was considered present if I^2^ > 50%. Random-effects models were used in this meta-analysis. Publication bias was assessed by searching trials that had been registered on ClinicalTrials.gov and World Health Organization International Clinical Trials Registry Platform but had not been published. A funnel plot was not used as < 10 studies were included for each outcome.

## Results

### Literature search

**Fig. 1:**
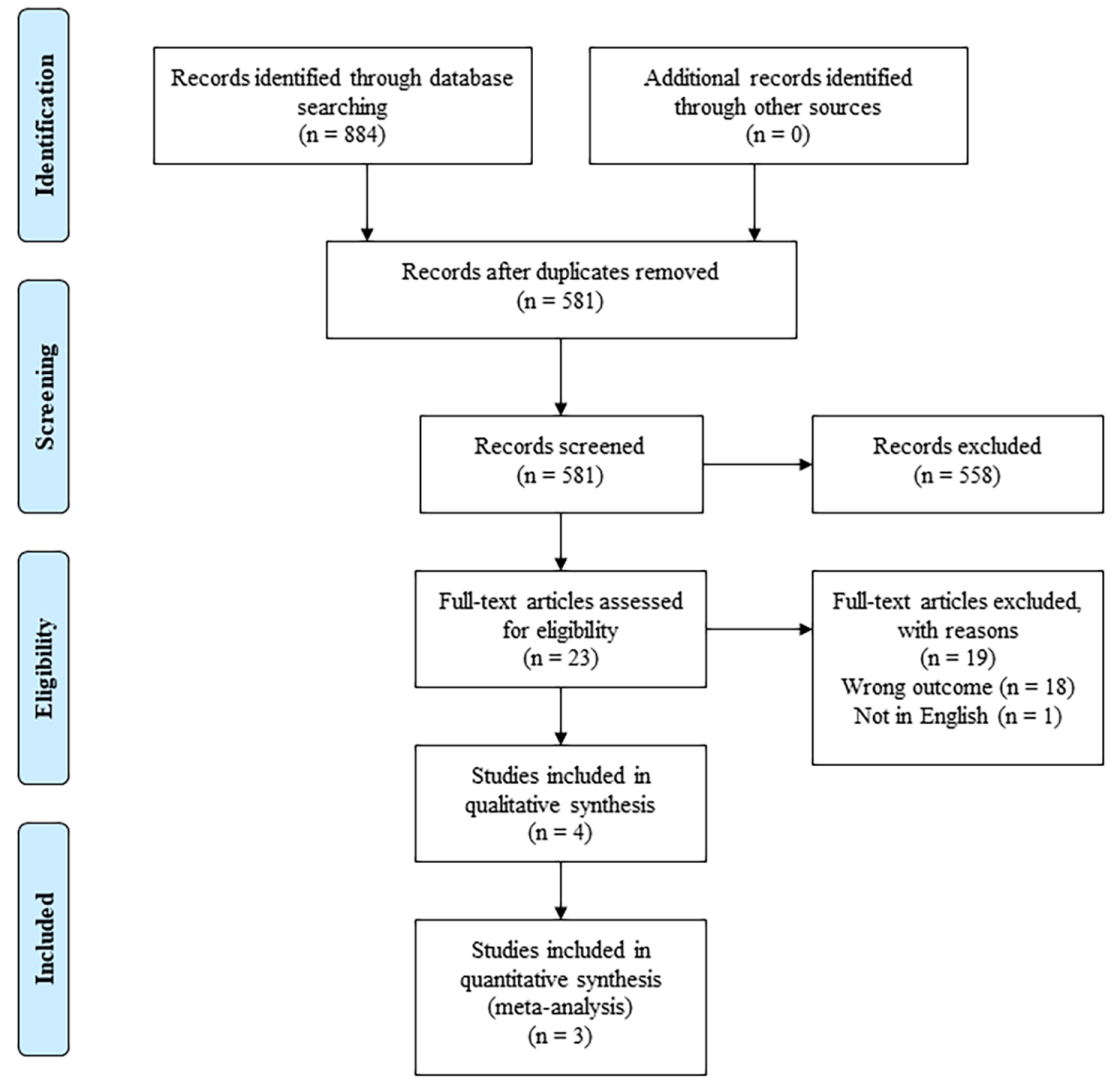
Flow diagram of literature search

After removing duplicates, 581 articles were identified. Following exclusion, 23 studies were screened for eligibility using full texts. Four studies [20–23] were finally included in the qualitative synthesis. The literature search and study selection processes are presented in Fig. 1.

### Study characteristics

The main characteristics of the included trials are summarized in Table 1. The four included studies involved 1268 central venous catheterizations: 633 ultrasound-guided catheterizations and 635 anatomical landmark-guided catheterizations. All four studies reported the incidence of CRBSIs, and one study [20] reported the incidence of catheter colonization for ultrasound-guided central catheter insertion and anatomical landmark-guided insertion. One study [22] did not describe sterilization and other three studies used povidone-iodine. Only one study [23] reported that the catheter insertion days were 10.1 ± 5.8 and 10.5 ± 5.2 (mean ± standard deviation) days in ultrasound-guided and landmark-guided groups, respectively. Only one study [23] reported the CRBSIs as the primary outcome. CRBSIs were defined according to the Centers for Disease Control and Prevention [24] in the two studies [21, 23]. CRBSIs were not defined in the other two studies [20, 22].

**Table 1:** Main characteristics of the included studies

Study	Country	Age (years, mean ± SD)	Population	Sample size	Outcome	Sterilization	Vein	Operator
Airapetian2013 [20]	France	US: 63 ± 15 LM: 67 ± 16	Adult	74	CRBSIs, catheter colonization	Povidone-iodine	Internal jugular or femoral	Ten residents
Dolu2015 [22]	Turkey	US: 53.6 ± 5.8 LM: 53.2 ± 9.10	Adult	100	CRBSIs	Not described	Internal jugular	Four residents
Gok2013 [23]	Turkey	US: 48.9 ± 21.9 LM: 51.8 ± 21.3	Adult	194	CRBSIs	10% povidone-iodine	Internal jugular	One anesthesiologist
Karakitsos2006 [21]	Greece	US: 58.3 ± 10.3 LM: 59 ± 9.5	Adult	900	CRBSIs	Povidone-iodine	Internal jugular	Attending cardiologists, intensivists, and surgeons

**US**: ultrasound-guided insertion; **LM**: landmark-guided insertion; **CRBSIs**: catheter-related bloodstream infections

### Risk of Bias in the included studies

**Fig. 2:**
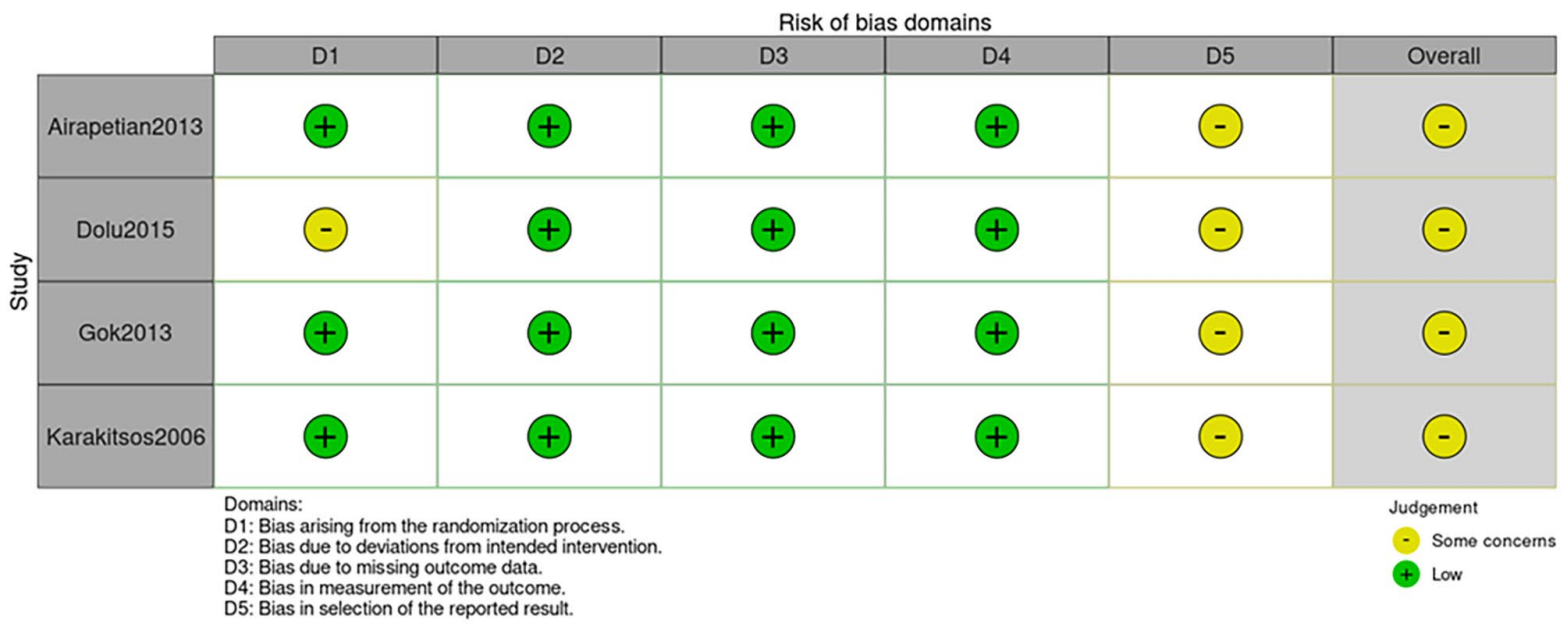
Risk of bias summary

The summary of the risk of bias are shown in Fig. 2. Overall, the four trials were categorized as having some concerns.

### Primary outcome

**Fig. 3:**
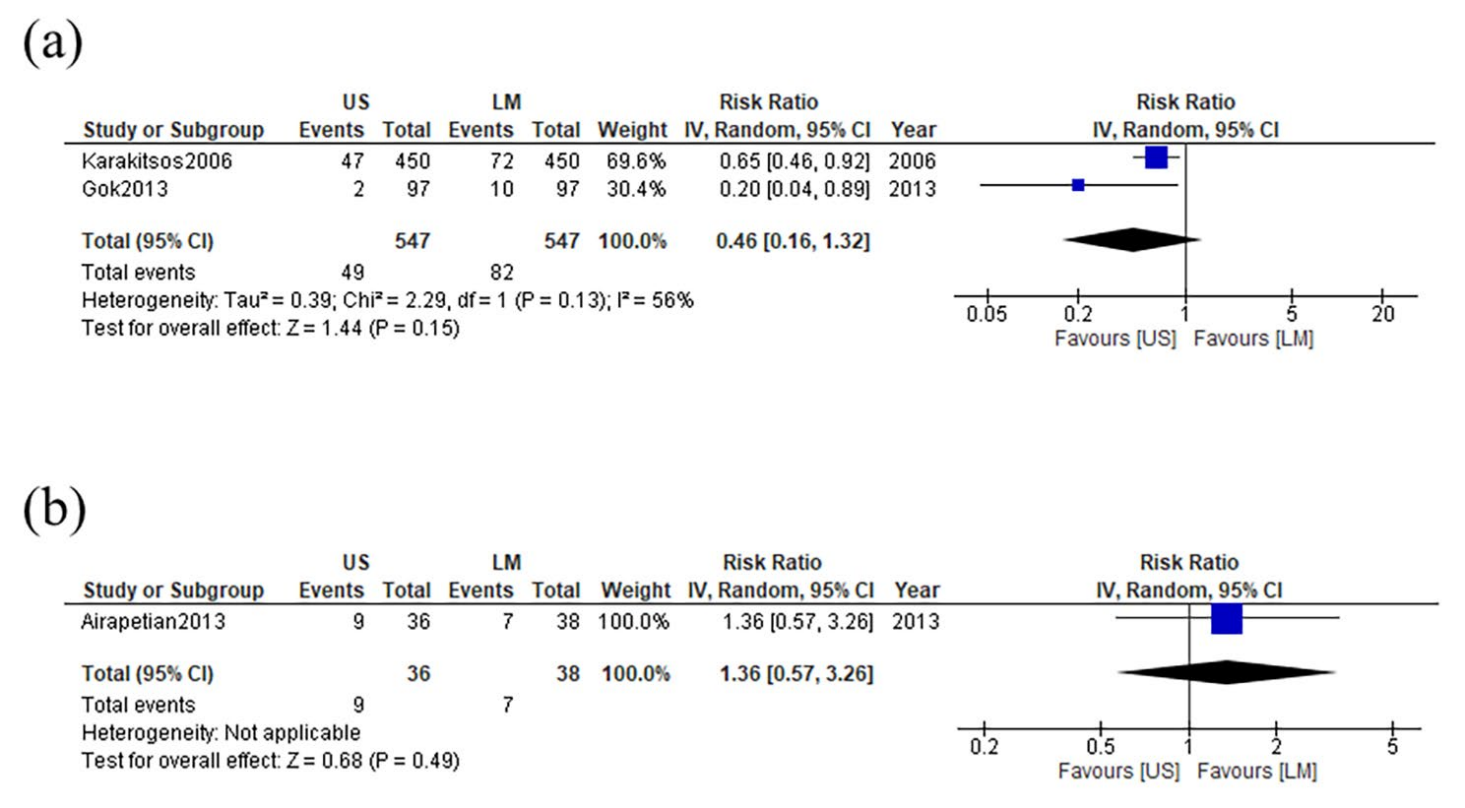
Forest plot comparing the incidence of (a) CRBSIs and (b) catheter colonization for ultrasound-guided versus anatomical landmark-guided central venous catheterization CRBSIs, catheter-related bloodstream infections; US, ultrasound-guided insertion; LM, landmark-guided insertion

All four trials that were included reported the difference in the incidence of CRBSIs between the ultrasound-guided and anatomical landmark-guided insertion techniques for central venous catheterization. Two studies had no events in either arm; therefore, we excluded these from the meta-analysis according to the Cochrane Handbook for Systematic Reviews of Interventions version 6.3. After pooling the trials, the ultrasound-guided insertion technique was associated with a slightly lower incidence of CRBSIs than the anatomical landmark-guided insertion technique (RR, 0.46; 95% confidence interval (CI), 0.16–1.32; p = 0.15 [Fig. 3**(a)**]). The absolute effect of ultrasound guidance in preventing CRBSIs was 81 fewer per 1000 (from 126 fewer to 48 more) patients, as a point estimate. Significant heterogeneity was observed among the included studies with respect to CRBSIs (I^2^ = 56%). The evidence summary is shown in Table 2. The microbiology data of the two included studies is shown in **Supplemental Table 1**.

**Table 2:** Evidence summary

Assessment of certainty							No. of patients		Efficacy		Certainty of the evidence	Importance
**No. of studies**	**Study design**	**Risk of bias**	**Inconsistency**	**Indirectness**	**Imprecision**	**Others**	**CRBSIs**	**Control**	**Relative index** **(95% CI)**	**Absolute index** **(95% CI)**		
**CRBSIs**												
2	RCT	Not serious	Not serious	Not serious	Serious ^a^	None	49/547 (9.0%)	82/547 (15.0%)	RR 0.46 (0.16 to 1.32)	81 fewer per 1,000 (126 fewer to 48 more)	⊕⊕⊕◯ Moderate	Critical
**Catheter colonization**												
1	RCT	Not serious	Not serious	Not serious	Very serious ^b^	None	9/36 (25.0%)	7/38 (18.4%)	RR 1.36 (0.57 to 3.26)	66 more per 1,000 (79 fewer to 416 more)	⊕⊕◯◯ Low	Important

**CI**: confidence interval; **RR**: relative risk; RCT: randomized controlled trial; **CRBSIs**: catheter related blood stream infections

**Explanations**

a. The sample size of 1094 met the optimal information size (OIS), however, the 95% confidence interval included both clinically meaningful thresholds for benefit and harm

b. The sample size of 74 did not meet the optimal information size (OIS), and the 95% confidence interval included both clinically meaningful thresholds for benefit and harm

### Secondary outcome

One of the four included trials reported the incidence of catheter colonization in both the ultrasound-guided and the anatomical landmark-guided insertion techniques for central venous catheterization. The ultrasound-guided insertion technique was not associated with a lower incidence of catheter colonization than the anatomical landmark-guided insertion technique (RR, 1.36; 95% CI, 0.57–3.26; p = 0.49 [Fig. 3**(b)**]). The evidence summary is shown in Table 2.

## Discussion

This meta-analysis of randomized controlled trials compared the efficacy of ultrasound-guided and anatomical landmark-guided central venous catheterization with respect to CRBSIs as well as catheter colonization. From the available data, this study suggests that the ultrasound-guided insertion technique might be associated with a lower incidence of CRBSIs than anatomical landmark-guided insertion techniques.

Numerous studies have compared the outcomes of insertion success and early complication rates between ultrasound-guided and anatomical landmark-guided central venous catheterization. However, most studies did not focus on CRBSIs, which have been reportedly associated with increased mortality [4–6], and patients would certainly benefit from reducing the incidence of this complication. In our literature search, only four randomized controlled trials compared the outcome of CRBSIs between ultrasound-guided and anatomical landmark-guided central venous catheterization. Two studies [20, 22] were excluded from the meta-analysis because there were no events in either arm, and this meta-analysis did not show the efficacy of ultrasound-guided central venous catheterization on the incidence of CRBSIs. Although the point estimate of RR is 0.46, the wide CI including 1 suggests the decreased certainty. Regarding catheter colonization, only one study [20] was included in the meta-analysis, and no association was observed between ultrasound-guided central venous catheterization and catheter colonization.

Two observational studies have compared the incidence of CRBSIs between ultrasound-guided and anatomical landmark-guided central venous catheterization; however, the difference was not statistically significant [25, 26]. Buetti et al. performed a post hoc analysis of three randomized controlled trials and demonstrated that the ultrasound-guided insertion technique was associated with an increased risk of CRBSIs (hazard ratio, 2.21; 95% CI, 1.17–4.16; p = 0.014) [15]. In that study, uncertainty about ultrasound techniques, including hygiene compliance, was stated as a limitation. Furthermore, as these studies randomized the patients according to the catheter insertion sites, skin asepsis, and dressings and not the insertion technique, the results may have been influenced by several confounding factors, especially because ultrasonographic guidance tends to be used in difficult or severe cases.

Regarding catheter insertion sites, one multi-center randomized controlled trial reported that the incidence of catheter colonization was higher in the femoral vein than in the internal jugular vein, while the incidence of CRBSIs was not different between the two veins [27]. Of the four studies included in our meta-analysis, one study adopted the internal jugular or femoral vein and patients were stratified according to the insertion site. In the three other studies, the insertion site was the internal jugular vein. Therefore, the insertion site itself is unlikely to have had much effect on the results.

This study had some limitations. First, only two studies and a relatively small number of patients were included in this meta-analysis, and significant heterogeneity was observed among the included studies on CRBSIs. The results of this study should be interpreted with caution. Second, only one included study focused on CRBSIs as a primary outcome. Third, only critically ill patients were included in the study. Finally, all the included studies detailed the use of povidone-iodine and not chlorhexidine for sterilization; therefore, caution should be exercised when extrapolating to the current practice of central venous catheterization.

## Conclusion

In conclusion, ultrasound-guided central venous catheterization might reduce the incidence of CRBSIs. However, only four studies were included in this systematic review. Additional randomized controlled trials are necessary to evaluate the effect of ultrasound-guided central venous catheterization on the incidence of CRBSIs and catheter colonization.

## Electronic supplementary material

Below is the link to the electronic supplementary material.

Supplementary Material 1

## Data Availability

All data generated or analyzed during this study are included in this published article and its supplementary information files.

## List of Abbreviations

CRBSIs: catheter-related bloodstream infections
CENTRAL: Cochrane Central Register of Controlled Trials
RR: risk ratio
CI: confidence interval
