# Supplementary material for: Incidence of catheter-related bloodstream infections following ultrasound-guided central venous catheterization: a systematic review and meta-analysis
Source: BMC Infect Dis. 2022 Oct 4;22:772. doi: 10.1186/s12879-022-07760-1 (PMC9533546; doi:10.1186/s12879-022-07760-1)
Supplement: Supplementary file 1 — Supplementary Material 1 [file 12879_2022_7760_MOESM1_ESM.docx]

**Supplemental Table 1**

The microbiology data of the included studies. (a) Karakitsos 2006, (b) Gok 2013

(a)

|  | US (n=47) | LM (n=72) |
| --- | --- | --- |
| Coagulase-negative staphylococci | 48.6% | 56.8% |
| *Staphylococcus aureus* | 27.0% | 24.1% |
| Enterococus species | 13.5% | 10.3% |
| *Escherichia coli* | 2.7% | 3.4% |
| Enterobacter species | 2.7% | 1.7% |
| *Pseudomonas aeruginosa* | *2.7%* | 1.7% |
| Candida species | 2.7% | 1.7% |

(b)

|  | US (n=2) | LM (n=10) |
| --- | --- | --- |
| *Staphylococcus aureus* | 0% | 30% |
| Coagulase-negative staphylococci | 0% | 40% |
| Enterococus species | 50% | 0% |
| *Klebsiella pneumoniae* | 50% | 20% |
| *Acinetobacter baumannii* | 0 | 10% |

US, ultrasound-guided insertion; LM, landmark-guided insertion
